# Dr. Koch's Consumptive Cure

**Published:** 1890-11

**Authors:** 


ARTICLE II.

### DR. KOCH'S CONSUMPTIVE CURE.

The article by Professor Koch in the Deusche Medinizische Wochenschrift, (German Medical Weekly,) published at Berlin, is entitled "Further communications on the cure of tuberculosis and experiments which Dr. Libbertz and Staff Surgeon Pruhl performed relating thereto under Professor Koch's direction."

In this article Professor Koch says that he is as yet unprepared to indicate the source from which the curative matter is derived. Neither is he ready to explain the method of preparation. The reason he gives is that the experimental work has not yet been brought to completion. He states, however, that the curative lymph itself can now be obtained from Dr. Libbertz, whose address is No. 28 Luenburger strasse, Berlin.

The lymph is described as consisting of a brownish transparent liquid. It is so prepared as to be proof against deterioration. When, however, it is diluted with water to the necessary degree for use the matter is liable to decay. It is necessary, therefore, that the attenuations should be perfectly sterilized by heat and preserved in wadding covering, or prepared with a solution of phenol fifty percentage strong.

When taken into the stomach the curative matter proves to have no effect. It must be applied sub-cutaneously by means of a valveless syringe. The kind of syringe recommended by Professor Koch is one furnished with a small, hollow rubber ball. This syringe approved itself to him during his bacteriological experiments. Its merit is that it can be easily and surely rinsed with absolute alcohol and kept in a perfectly aseptic condition. In thousands of cases, he says, where it has been used for subcutaneous injections not a single abscess resulted.

When the curative matter is applied to a patient the usual course is to inject it under the skin of the back, between the shoulder blades and in proximity to the loins. The experiments show that human beings are much more susceptible to the effect of the new substance than are guinea pigs, which have been largely used in the course of the investigations. Two cubic centimetres of the fluid applied to a guinea pig produced little, if any, apparent effect. Twenty-five hundredths of a cubic centimetre, however, intensely affected a healthy, full-grown man who was subjected to experiment.

Prof. Koch experimented with the fluid upon his own body and describes the effect. He injected twenty-five hundredths of a cubic centimetre of the fluid under the skin of his upper arm. Three or four hours after the injection was made he experienced a contraction of the limbs and a marked feeling of lassitude. At the same time he felt a desire to cough, together with difficulty of breathing. These symptoms increased rapidly and in the fifth hour he experienced an unusually violent rigor. The shivering lasted for nearly an hour and was accompanied with nausea and vomiting. The temperature of his body rose to 39.6 centigrade. After a period of twelve hours the symptoms began to abate, the temperature of the body declined and on the following day resumed its normal degree. The heaviness of the limbs and the feeling of lassitude, however, continued for some days, during which time the point on his arm at which the injection was made continued to be painful and remained red.

The experiments so far conducted show that the lowest limit of effective strength of the fluid in a healthy human body is one hundredth of a cubic centimetre. When this amount is applied to a healthy human subject it produces little or no reaction. The same result follows when fluid of this strength is applied to diseased persons who are suffering from other than tuberculous affections, but in persons affected with tuberculosis the same quantity produces a strong general and local reaction. The general reaction consists of an attack of fever, which usually begins with shivering; the temperature of the body rises to over 39 and in some instances even to 41 centigrade. At the same time pains in the limbs are noticeable. The patient coughs, experiences much irritation and great exhaustion. Some patients also suffer from nausea and vomiting. In some cases there is noticed a slight icteric (jaundice like) coloring, or exanthemia, resembling measles, on the chest or neck. The symptoms just described begin to manifest themselves four or five hours after the injection of the curative substance. They last from twelve to fifteen hours. The patient is not much affected by the attack induced by the fluid and after it is over feels comparatively well, even better in fact than before the injection.

The second or local reaction produced by the injection of the fluid in a patient suffering from tuberculosis can best be observed in persons whose tuberculous affections are visible as, for instance, in the case of persons suffering from lupus. The changes which ensue in these cases show, in a surprising manner, the specifically anti-tuberculous effect of the remedy. Within a few hours after an injection of the fluid has been made under the skin of the back, the lupus sores begin to swell and redden. During the fever which the patient experiences as already described, the swelling and reddening of the sores increase until finally the lupus tissue assumes in places a dark brown tint and a necrotic condition. After the subsidence of the fever, the swelling of the lupus sores gradually decreases and possibly disappears altogether within two or three days. Meanwhile, however, the lupus centres have become covered with an incrustation, formed by exuding serum, which dries up as it reaches the air. These incrustations gradually form into scabs, which fall off after two or three weeks. In some instances the effect just described is produced after a single injection of the curative matter, when a red, smooth scar is left. These changes are absolutely limited in extent to such portions of the skin as are clearly recognized as lupus. In tuberculosis of the lymphatic glands, bone articulations, etc., these local reactions are less striking, but are still clearly perceptible to the eye and touch. In these cases there is swelling of the parts affected, and more pain than lupus patients suffer, while in the parts adjacent to the diseased centres the surface also becomes red.

The reaction produced in the internal organs, especially the lungs, when the curative substance is injected, is not of course open to observation apart from the increased expectoration and cough. In all the experiments after the first injections if any tuberculous process existed in the body the appearances already described supervened. Absolutely no exception was noted whenever a dose amounting to one-hundredth part of a cubic centimetre of the substance had been applied. One may assert with confidence, therefore, that the remedy may be considered an indispensable auxiliary to diagnosis, and in doubtful cases incipient phthisis can positively be diagnosed by its use, even if positive information of the nature of the disease cannot be obtained by the discovery of bacilli, or of elastic fibres in the sputum, or by a physical examination.

Tuberculous affections of the glands, latent tuberculosis of the bones, doubtful skin tuberculosis, &c., can by the use of this fluid be easily and certainly diagnosed. Moreover, in cases of lung or joint tuberculosis which have apparently passed off it will be possible, by the use of the new substance to ascertain with certainty whether the morbid process is really and absolutely ended.

Professor Koch expresses the belief that his remedy will certainly prove a cure for incipient phthisis. Whether, however, the cure will be final or definite has not, he says, been clearly proved. Further experiments and continued use of the remedy will be necessary to determine this question.

The curative properties of the new remedy, Professor Koch declares, are of still greater importance for diagnosis. What the fluid kills is not tubercular bacillus, but the tubercular tissue. This fact indicates the well-defined limits which the efficacy of the remedy will be able to reach. In other words, it can only influence living tubercular tissue. It has no effect whatever upon dead tissue, such as decayed caseous matter, necrotic bones and the like. More than this, it produces no effect upon tissues which have already been killed by the application of the remedy. It is quite possible that such dead tissue may still contain living tuberculous bacilli. These may then be either expelled with the necrotic tissue, or it may be that under special circumstances they may again invade adjacent living tissues.

It follows, therefore, that tuberculous tissue that is still living must first be made to decay. When this has been accomplished every effort must be made to remove the dead matter by surgery. In cases where this method is impossible and secretion can only slowly proceed by the self-help of organism threatened, the living tissue must at the same time be protected by continual applications of the remedy so as to guard against the reimmigration of the parasites.

The fact that the remedy causes decay only of tuberculous tissues affecting exclusively living tissues explained why the curative fluid may be applied in rapidly increased doses.

After the patient has been under treatment for a period of three weeks a dose of the fluid of 500 times the strength of the original dose can be applied, for at the beginning, when a large quantity of living tuberculous tissue exists, a small quantity of the remedy suffices to produce a strong reaction, but each succeeding injection causes the disappearance of a certain quantity of tissue capable of reaction. It naturally follows, therefore, that increased doses are necessary to obtain the same degree of reaction. As soon as patients treated with increased doses experience as little reaction as non-tuberculous people it may be assumed that all tuberculous tissue open to reaction is dead. Whether this conclusion is correct, time will show. For the present, however, Professor Koch is convinced that this conclusion is a sound one.

In all cases of lupus so far treated the amount of fluid injected has been in the first instance one-hundredth part of a cubic centimetre. Time has been allowed for the reaction to take its full course. After a period of from one to two weeks a second injection of the same amount has been made. This treatment has continued until the reaction grew weaker, and finally ceased. In two cases of lupus of the face the sores after three or four injections became scarred and presented a smooth surface. Each one of these cases has suffered for many years, and had been treated by other methods without beneficial result.

Persons suffering with tuberculosis of the lymphatic glands, bones or joints have been treated with precisely the same success. There has been rapid healing in the milder cases and cases of recent development of the disease, while in several cases the improvement, while slower, has been steady. Patients with pronounced tuberculosis of the lungs have proved far more susceptible to the remedy than those suffering with surgical tubercular affections. Consumptives have in almost every instance manifested a strong reaction on greatly reduced doses. With such patients, therefore, a beginning should be made with doses of two one-thousandths part of a cubic centimetre, or even with one one-thousandth part. From this small incipient dose one can advance to such quantities as the patient can easily bear. In the experiments that have been conducted consumptives have accordingly first received a sub-cutaneous injection of one one-thousandth part of a cubic centimetre.

Then, when the temperature increased, the same dose was applied daily until no further reaction occurred. Thereupon the dose was doubled, two one-thousandths part of a cubic centimetre being injected regularly until again no reaction occurred. This method was continued with almost always an increase of one-one-thousandth part of a cubic centimetre, or at most two one-thousandths up to one one-hundredth part, and so on upward. In this way the patient would be brought to take very high doses almost without fever and almost imperceptibly to himself. Consumptive patients who are still fairly strong reach increased doses much more quickly, and favorable results follow with corresponding rapidity. As a general rule the coughing and expectoration are increased somewhat after the first injections. Then they become gradually less, and in the most favorable cases will ultimately wholly disappear.

In the cases experimented upon under the direction of Professor Koch the expectorations gradually lost their purulent property and assumed a mucous character. The number of bacilli expelled usually decreases only when the expectorations begin to assume the mucous appearance. The bacilli then disappear entirely for a time, but on occasions again appear until expectoration totally ceases. At the same time the night sweats cease, the patients begin to look better and to increase in weight. Patients who have been treated in the early stages of phthisis have all been freed from morbid symptoms within from four to six weeks, when they may be regarded as healed.

Consumptives with large cavities in their lungs will probably only experience benefit from the new remedy in exceptional cases, though most cases show temporary improvement.

Professor Koch deprecates the mechanical and indiscriminate application of the remedy. He holds that it would be preferable that the treatment should be applied in suitable institutions where careful observations would be possible.

Professor Koch says that sufficient experience has not yet been collected regarding tuberculosis of the brain and the larynx and miliary tuberculosis to justify the expression of any opinion in regard to the efficacy of the remedy.

In all cases Professor Koch emphasizes the necessity of early treatment. Only in incipient stages of the disease, he declares, can the remedy fully develop its efficacy.

The Frank Courier states that the lymph used by Professor Koch for the cure of tuberculosis is prepared in an incubating stove within a space that is hermetically sealed and sterilized and thereby rendered free from fungus. The interior of the air-tight space is divided by an unglazed porcelain diaphragm into an upper and lower section. In the upper section is placed a salted meat broth in a gelatinous state containing colonies of the tubercule germ. This mass gradually liquefies and the gelatine liquid drops slowly through the porcelain plate into the lower section. The liquid then contains all the secretory products, but is free from all living or dead germs or reproductive spores, and is the lymph as used.

By the injection of the lymph the tubercule germ is killed, and at the same time the injected particles retain sufficient strength to detach and expel the dead germs, together with the dead tissue. The reparative process ensues and healing follows.

A meeting of the Medical Society was recently held, at which Professor Virchow, the celebrated German physician, presided. Professor Virchow made an address, in which he defended Professor Koch against the charge of having prematurely published the facts regarding his discovery. The first information he gave regarding the subject, Professor Virchow said, was given to the Medical Congress recently held in Berlin, and the disclosures he then made were in compliance with the urgent request of a committee of the congress and Dr. von Gossler, Prussian minister of ecclesiastical affairs, education and medicinal affairs.

Empress Frederick gave an audience to Professor

Koch, who explained to her Majesty the results already obtained from the use of his curative lymph and the benefits he hoped would be conferred upon humanity by its general use.

The Deutsche Medicinische Wochenschrift contains a report of Drs. Bergmann, Frantzel and others on Koch's treatment of tuberculosis. The article, which is written in scientific language, adds little to what is already known. Dr. Frantzel believes that advanced cases need a long course of treatment before the efficacy of the remedy can be shown. He agrees with the other doctors that the greatest value of the remedy is as a means of diagnosis and in the treatment of tubercular skin diseases. In disease of internal organs the remedy is of doubtful efficacy, where cavities are small they may cicatrize, but where they are large, owing to the flow of matter and the presence of other micro-organisms, the wasting process continues and death ensues.

Many foreign doctors have issued warnings to consumptives against undertaking a dangerous journey to Berlin in winter. It is better, they say, for patients to wait for the arrival of the lymph at home, where they can obtain quieter and more careful treatment than is possible in Berlin, which is now crowded with cases.

The Nachrichten says that Emperor William has bestowed the grand cross of the Order of the Red Eagle upon Professor Koch.

Three patients treated by the Koch method are reported dead. They were all in a critical condition before they received the injections.

Physicians are agreed that Dr. Koch's tuberculosis remedy proves effective only in the treatment of mild cases of the disease.

The municipal authorities have resolved to confer the freedom of the city upon Dr. Koch. They have also voted funds and appointed a committee to provide a temporary hospital for his tuberculosis patients and accommodations for his experiments. This measure is taken pending government action in the matter.

